# Multiple channels, low-cost, and dual data storage data logger for building a soil temperature network

**DOI:** 10.1016/j.ohx.2024.e00582

**Published:** 2024-09-11

**Authors:** Gustavo N. Santiago, Ignacio Ciampitti

**Affiliations:** a Kansas State University, Department of Agronomy, Manhattan, KS, USA; b Kansas State University, Institute for Digital Agriculture and Advanced Analytics, Manhattan, KS, USA

**Keywords:** Temperature sensor, Data logging, Modular, Remote storage

## Abstract

Temperature measurement is critical in many areas of research, particularly in agriculture, where it can have a significant impact on crop health and yield. Experiments such as seed germination often require numerous temperature sensors to collect extensive data. Typically, data loggers are used to store information, but market options are expensive and offer limited, non-customizable inputs (channels), creating challenges for comprehensive soil temperature monitoring. This study aims to develop a network of open-source, low-cost data loggers with multiple customizable channels for local and remote temperature data storage. The hardware includes Arduino, temperature sensors, a Real Time Clock, and a LoRa module to transmit data to a LILYGO TTGO board, which sends it to a remote MongoDB database while also storing it locally on a microSD card. In addition, a digital tool was developed to retrieve and display both current and historical readings from the MongoDB database. The total cost of this hardware is approximately US$ 72 (based on current prices) for the simplest network, which is approximately 18 % of the commercial cost. The system achieved a root mean square error (RMSE) of 1.6 °C compared to a manual sampling probe thermometer, proving it to be a reliable measurement source. The hardware developed in this study surpasses commercial options by allowing the integration of multiple sensors and emitters, creating a network of data loggers at a lower cost. In addition to the hardware, an open-source digital tool was developed to visualize historical data at no additional cost.

## Graphical abstract

**Figure:**
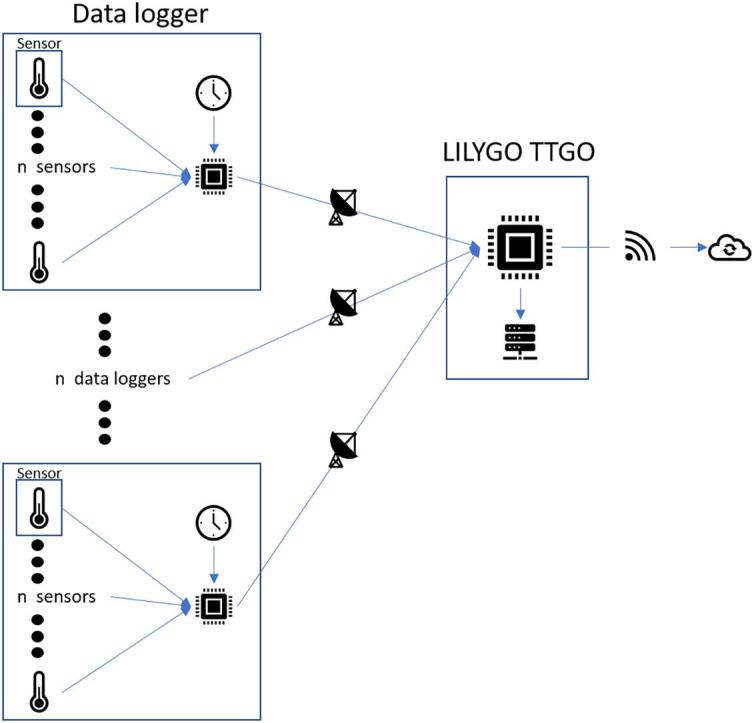

## 1 Specifications table

**Table:** 

Hardware Name	Temperature data logger
Subject area	Environmental, planetary, and agricultural sciences
Hardware Name	Field measurements and sensors
Closest commercial analog	Wifi Thermocouple Data Logger
Open source license	GPL General Public License
Cost of Hardware	Minimum of U$72
Source file repository	Mendeley Data (https://data.mendeley.com/datasets/czgpmp8cr7/2)

## 2 Hardware in context

Temperature is a critical factor in many research areas, particularly in agriculture, due to its direct impact on crop health and yield [1], [2]. Investigation of temperature effects is essential for understanding its impact on crop systems [3], [4]. In particular, assessment of soil temperature is vital due to its influence on germination, root development, and microbial activity, all crucial for healthy plant growth [5], [6], [7]. Additionally, soil temperature affects nutrient availability and uptake, impacting overall yield [8], [9]. By monitoring soil temperature, farmers can optimize planting times, irrigation, and fertilization schedules, leading to more sustainable agricultural practices [10], [11], [12]. Soil temperature monitoring also aids in root disease research and improves crop protection management techniques [13], [14].

In experiments requiring extensive temperature data, mainly in greenhouses and growth chambers, such as seed emergence studies, numerous sensors are needed to obtain comprehensive information [15]. Data loggers, which record temperature along with date and time, are commonly used for such investigations [16]. However, commercially available data loggers have some limitations, addressed by Eze et al. [17], which include a lack of long-range wireless data transmission capabilities, reduced memory storage capacities, and the capacity to read and record many data at the same time. Gandra et al. [18], while developing a data logging system for ecological applications also observed limitations on power consumption and, consequently, autonomy. Another limitation includes the limited number of non-customizable channels for temperature data and high unit cost [19]. When researchers need more sensors than a data logger can accommodate, additional units must be purchased, thus increasing the research budget. The costs multiply further if the research involves different blocks or larger field-scale studies requiring a set of data loggers [20]. These cost constraints to deploying a network of data loggers, which can often result in incomplete datasets that impact continuous monitoring and research studies [18].

These data loggers typically do not interconnect, storing data locally on the device, necessitating laborious and time-consuming data manipulation to combine collected data from multiple loggers. Commercial data loggers usually store data on local databases, such as Secure Digital (SD) cards. While locally stored data is reliable, accessing measurements from distant experimental sites can be cumbersome and time-consuming [21]. Some commercial data loggers can upload data to remote databases, but these tend to be more expensive and lack of open-source flexibility, preventing user modifications [22], [23].

Considering these challenges, some attempts have been made to develop hardware that addresses specific needs. Gandra et al. [18] developed a low-cost and versatile data logging system for environmental applications at a cost of €150 using Arduino, a real-time clock, and an SD memory card, a reduced amount compared to commercial versions. Hernández-Rodríguez et al. [24] demonstrated the possibilities of using low-cost and multi-purpose data loggers for monitoring air quality, human activity, motion, and exhaust gas monitoring, focusing on robustness, response time, replicability, calibration, and similarity. A system for storing data collected for solar thermal collectors in the cloud and displaying it to the user has been developed by Panagopoulos & Argiriou [25], illustrating attempts performed to move data storage to remote systems. However, to the best of our knowledge, no low-cost network of multi-channel data loggers storing data in local and remote systems has been developed so far.

Following this rationale and aiming to fill this research gap, the objective of this study is to develop a network of open-source, low-cost data loggers with multiple customizable channels for storing temperature data in both local and remote databases. A secondary objective is to develop a digital tool to visualize current and historical readings without maintenance or usage costs.

## 3 Hardware description

**Fig. 1:**
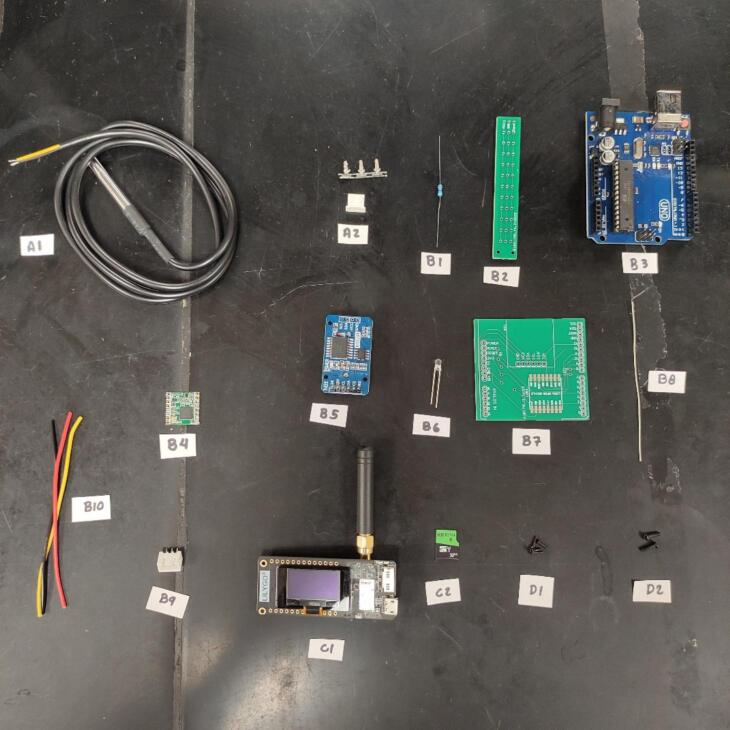
Parts described in Table 1 with the labels.

**Fig. 2:**
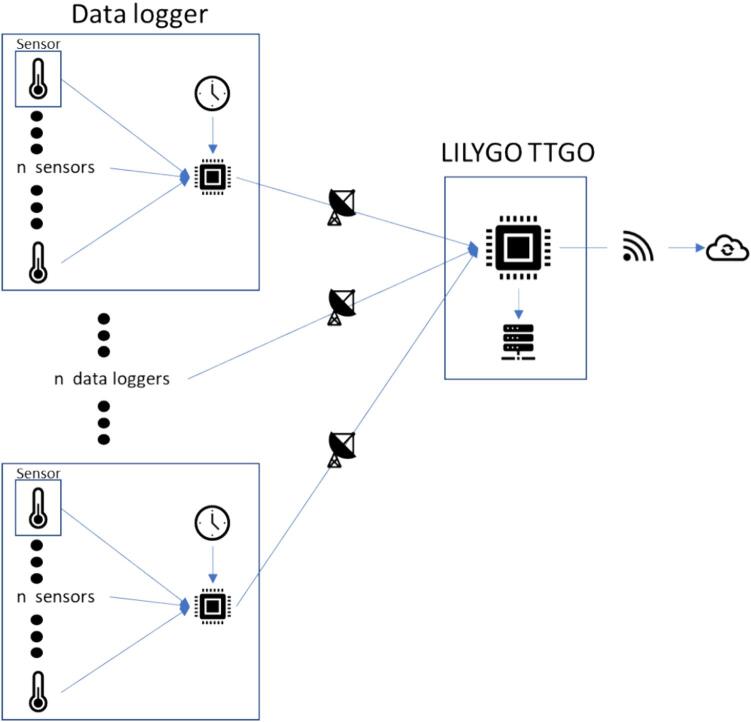
Flowchart illustrating the operational processes of the temperature data loggers’ network.

The hardware, basic design of the data logger, is presented in Table 1. A visualization of the hardware is presented in Fig. 1. The DS18B20 Temperature sensor(s) (A1) is responsible for reading the temperature data. More than 1 temperature sensor can be used at the same time, for that, it is necessary to attach the temperature sensors to a Printed Circuit Board (PCB) (B2) designed exclusively for this purpose. This PCB is then connected to the Arduino Uno (B3) through wires. The DS3231 RTC (B5) provides the date and time information. The date time, and temperature data, are then sent via LoRa using the RFM96 LoRa board (B4) to the LILYGO TTGO microcontroller (C1). A network is obtained by combining more than 1 data logger. When the LILYGO TTGO microcontroller receives the information, it stores it in the 32 GB microSD card (C2) in.txt format and uploads it to the cloud. To upload data to the cloud, the LILYGO TTGO must be connected to the internet through Wi-Fi and have an Application Programming Interface (API) and a MongoDB database running on a remote web server. If the researchers do not want to upload data to the cloud, they can store data only in the microSD card. A flowchart illustrating the process is presented in Fig. 2.

**Table 1:** Hardware required to build the network of temperature data loggers.

Label	Part Name	Description
A1	DS18B20 Temperature sensor	Obtain temperature measurements
A2	3 pin male JST-XH housing	Connect the sensor to the PCB
B1	4.7 k Ohm resistor	Control the flow of electrical current in the sensors
B2	Sensors PCB	Organize the sensors in one place
B3	Arduino Uno	Control data obtaining and emissions
B4	RFM96 LoRa board	Send the sensors data using LoRa communication
B5	DS3231 RTC	Obtain the date and time
B6	White 3 mm LED	Inform the operator that something is not right
B7	Arduino shield PCB	Organize the Arduino’s components in one place
B8	7.8 cm of 1 mm copper wire	Make the antenna
B9	3 pin female JST-XH housing	Connect the sensor to the PCB
B10	3 sets of copper wire	Connect the sensors PCB to the Arduino shield PCB
C1	LILYGO TTGO LoRa32 ESP32 SD Card Bluetooth	Receive the data, save in the microSD card and send to a remote database
C2	32 GB microSD card	Store the data
D1	M2 screws	Attach the Arduino shield PCB to the structure
D2	M1.7 screws	Attach the LILYGO TTGO and the sensors to the structure
D3	Pin male header	Connect the Arduino to the Arduino shield PCB

The DS18B20 temperature sensor was chosen for its affordability, waterproof options, wide range of temperature measurements (−55 °C to +125 °C), high accuracy (±0.5 °C), and easy of configuration and use. The Arduino Uno was chosen for its simplicity, popularity, and price, allowing users with little experience in hardware design to reproduce this study. The RFM96 LoRa board was used due to its low cost, diverse available tutorials that facilitate further customization, low power consumption, and long range communication. The LILYGOTTGO LoRa32 ESP32 SD Card was chosen because this board has embedded WiFi, Bluetooth, a microSD card slot, an LED display, and LoRa, and is very affordable for its specifications.

The data logger presented in this study offers the ability to include a high number of temperature sensors within a single unit. Commercial data loggers typically support up to 12 channels, with prices ranging from US$80 to US$350, excluding sensors. In contrast, our data logger supports up to 24 channels and costs a minimum of US$72, including one sensor. For studies requiring 24 temperature measurements, instead of purchasing at least two commercial data loggers (minimum cost of US$160) plus sensors, researchers can assemble our data logger for US$126. Furthermore, if an additional block is needed for an experiment, there is no need to spend US$80 to US$350 on a completely new system; instead, researchers can add a single data logger for only US$35 (current prices for July 2024). This cost efficiency increases with the scale of the study, making our data logger a more economical choice for extensive research.

The connectivity of our data logger is another significant advantage over commercial options. Data sent from the data loggers to the LILYGO TTGO via LoRa connection eliminates the need to place data loggers close to each other, allowing installation in different and distant locations. Additionally, our data logger can store data in two ways: locally on a microSD card or remotely in a MongoDB database. Commercial data loggers with remote data storage capabilities are significantly more expensive (starting at US$220) and offer a limited number of channels.

Finally, our data logger uses open hardware and open software, making it fully customizable to meet the specific needs of researchers.

Key benefits of our data logger network include:

• Reduced overall research costs,
• remote data transmission to a database, eliminating the need for physical presence in the sampling area to verify readings,
• inexpensive maintenance and repair, and
• fully customizable, allowing adjustments to meet the specific research needs.

## 4 Design files

All packages and files can be downloaded from the Mendeley repository, and these GitHub links: TempVis, Soil_Temperature_Datalogger, TempDataLogger_API with the description of each file provided in Table 2. The README section of these GitHub links provides supplementary code explanations and running instructions.

**Table 2:** Design file summary.

Design file name	File type	Open-source license	Location of the file
Visualization tool	R code (.R)	GNU General Public License	Mendeley Data (https://data.mendeley.com/datasets/czgpmp8cr7/2)
Code for the API	python code (.py)	GNU General Public License	Mendeley Data (https://data.mendeley.com/datasets/czgpmp8cr7/2)
Arduino emissor code	Arduino code (.ino)	GNU General Public License	Mendeley Data (https://data.mendeley.com/datasets/czgpmp8cr7/2)
LILYGO TTGO receiver code	Arduino code (.ino)	GNU General Public License	Mendeley Data (https://data.mendeley.com/datasets/czgpmp8cr7/2)
Arduino’s shield PCB	json file	GNU General Public License	Mendeley Data (https://data.mendeley.com/datasets/czgpmp8cr7/2)
Arduino’s shield PCB (for PCB manufacture)	gerber file	GNU General Public License	Mendeley Data (https://data.mendeley.com/datasets/czgpmp8cr7/2)
Sensor’s board PCB	json file	GNU General Public License	Mendeley Data (https://data.mendeley.com/datasets/czgpmp8cr7/2)
Sensor’s board PCB (for PCB manufacture)	gerber file	GNU General Public License	Mendeley Data (https://data.mendeley.com/datasets/czgpmp8cr7/2)
Both circuits schematic	json file	GNU General Public License	Mendeley Data (https://data.mendeley.com/datasets/czgpmp8cr7/2)
Arduino case	stl file	GNU General Public License	Mendeley Data (https://data.mendeley.com/datasets/czgpmp8cr7/2)
Arduino cover	stl file	GNU General Public License	Mendeley Data (https://data.mendeley.com/datasets/czgpmp8cr7/2)
LILYGO case	stl file	GNU General Public License	Mendeley Data (https://data.mendeley.com/datasets/czgpmp8cr7/2) and https://www.thingiverse.com/thing:6127782
LILYGO cover	stl file	GNU General Public License	Mendeley Data (https://data.mendeley.com/datasets/czgpmp8cr7/2) and https://www.thingiverse.com/thing:6127783

**Fig. 3:**
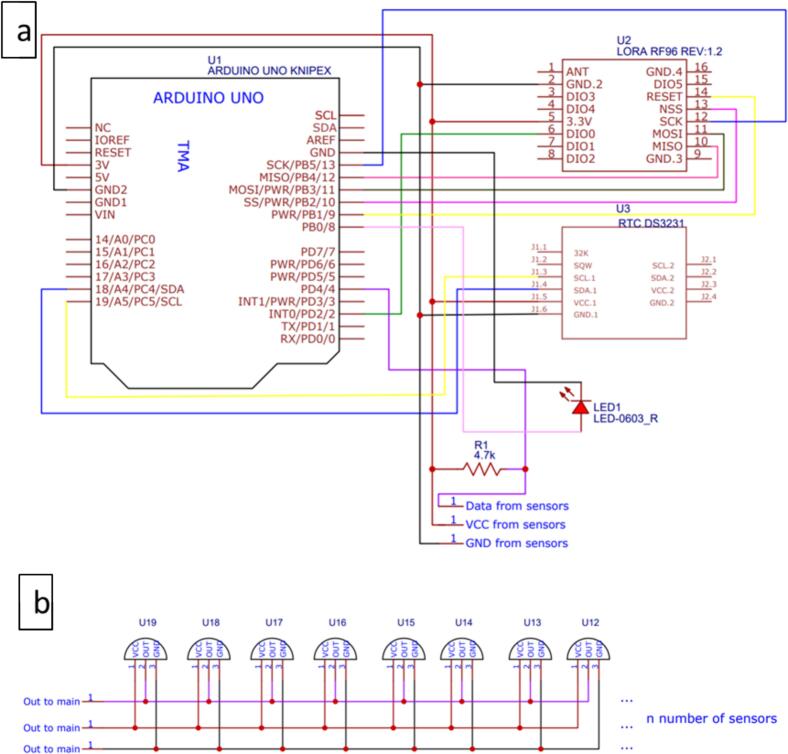
Arduino shield (a) and sensors’ board (b) schematics.

The board schematics for the Arduino Shield and the sensor board are shown in Fig. 3. Note that in the lower part of the Arduino shield schematic (Fig. 3a), some text informs that these wires come from the sensors board (Fig. 3b). In addition, note that the sensor board is designed to fit a custom number of sensors connected in parallel (Fig. 3b). The respective PCBs have a text layer to indicate where the parts must be placed, aiding in the assembly process.

**Fig. 4:**
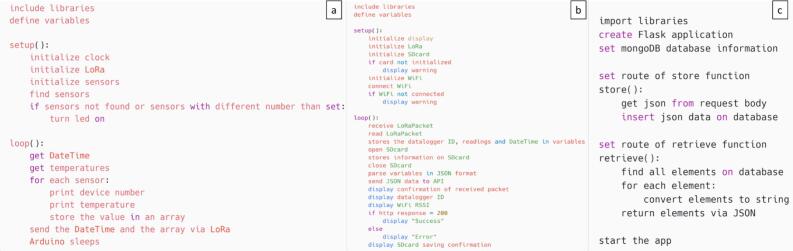
Pseudo-code for the data loggers (a), LILYGO TTGO (b), and the Python API (c).

The R [26] code in Table 2 is a digital tool that can be used to visualize the data collected by the data loggers in a more user-friendly format; the data can be later exported in CSV format for later analysis and manipulation by the researchers. The Arduino (.ino) codes must be modified to include the number of sensors used in each data logger, the internet and LoRa configurations, and the API’s URL. We provided files in the cases where the researcher desires to use both remote and local databases, only the local database or only the remote database. The pseudocode for the Arduino codes and the Python API is shown in Fig. 4. The pseudo-code for the data logger (Fig. 4a) initializes various components, reads sensor data, and transmits it via LoRa communication before entering a sleep state. The pseudo-code for the LILYGO TTGO (Fig. 4b) receives data via LoRa, stores it on an SD card, sends it to an API, and displays various statuses and confirmations. Lastly, the pseudo-code for the Python API (Fig. 4c) sets up a Flask application, configures a MongoDB connection, and defines two functions i) receives data via JSON and posts it to the database, and ii) retrieves all elements from the database and returns them as JSON; finally, it launches the Flask application.

In this study, we provided json files, which allow modifications in the PCB design or the schematics of the project, increasing the customizability and contributing to the open-source community. The gerber files are files ready to be sent to a PCB maker company to be manufactured. The stl files provided here can be used to print cases and protective structures in a 3D printer. The last two stl files were not developed by the authors but developed by the user “mason_swiss” and obtained through the open-source ThingIverse community.

## 5 Bill of materials

The table with the bill of a minimum data logger network is listed in Table 3. The starting cost of the data logger network presented in this study is US$72.3 (current prices for July 2024). This initial cost is the minimum required to build one data logger using one sensor. If more temperature sensors are required, the researchers have to spend an additional US$2.32 for each sensor. For each desired data logger, researchers will have to spend a minimum of US$35.4 in each additional unit, since it will not be necessary to buy another LLYGO TTGO and a 32 GB microSD card. In addition, this microSD card capacity is a suggestion and can be replaced by another microSD card with a lower price.

**Table 3:** Materials to build the sensor, components, units, cost per unit, total costs (price by July 2024), and sources.

Components	Number	Cost per unit (USD)*	Total cost (USD)	Source of materials
DS18B20 Temperature sensor + 4.7 k Ohm resistor	1	2.32	2.32	Gikfun DS18B20 Temperature Sensor Waterproof Digital Thermal Probe Sensor for Arduino (Pack of 5pcs) EK1083:Amazon.com: Industrial & Scientific
Arduino Uno	1	14.99	14.99	Amazon.com: ELEGOO UNO R3 Board ATmega328P with USB Cable(Arduino-Compatible) for Arduino: Electronics
RFM96 LoRa board	1	11.45	11.45	Amazon.com: RFM95W 915Mhz LoRaTM Wireless Transceiver, LoRa Ultra Long Range/Super Anti-interference/Low Current Consumption Wireless Receiver Transmitter Module: Electronics
DS3231 RTC	1	2.6	2.6	Amazon.com: HiLetgo 5pcs DS3231 AT24C32 Clock Module Real Time Clock Module IIC RTC Module for Arduino Without Battery: Industrial & Scientific
White 3 mm LED	1	0.07	0.07	Amazon.com: CHANZON 100 pcs 3 mm White LED Diode Lights (Clear Round Transparent DC 3 V 20 mA) Bright Lighting Bulb Lamps Electronics Components Indicator Light Emitting Diodes: Industrial & Scientific
LILYGO TTGO LoRa32 ESP32 SD Card Bluetooth	1	31.9	31.9	Amazon.com: LILYGO LoRa32 433Mhz ESP32 Development Board OLED 0.96 Inch SD Card BLE WiFi TTGO Paxcounter Module: Electronics
32 GB microSD card	1	4.99	4.99	Amazon.com: PNY 32 GB Elite Class 10 U1 microSDHC Flash Memory Card 3-Pack − 100 MB/s, Class 10, U1, Full HD, UHS-I, micro-SD: Electronics
Sensors PCB	1	1	1	You can use any PCB maker (PCB way, JLCPCB, ect)
Arduino shield PCB	1	1	1	You can use any PCB maker (PCB way, JLCPCB, ect)
3D printed parts	96 g	0.01599 per gram	1.54	Amazon.com: Blue ABS Filament 1.75 mm, NovaMaker Less Odor ABS 3D Printer Filament, Dimensional Accuracy +/- 0.03 mm, 1 kg Spool(2.2lbs): Industrial & Scientific
JST connectors	5	0.019	0.095	Amazon.com: Twidec/500PCS 2.54 mm JST Connector Kit with 2/3/4/5/6 Male and Female Pin Housing Connector Adapter Plug and 2.54 mm Female Pin Header Wire Terminal Connector Kit N-010-W: Electronics
Copper wire	35.5 cm	0.005	0.2	18 AWG Stranded Wire Kit – Silicone Coated Copper Wires 18 Gauge Pre-Tinned 16ft/5m Each Spool, 6 Colors (Black, Red, Yellow, Green, Blue, White), Electrical Jumper Wire Hook Up Wire Kit from Plusivo:Amazon.com: Tools & Home Improvement
Screws	6	0.027	0.16	Amazon.com: NINDEJIN 720pcs Laptop Notebook Computer Carbon Steel Screws Kit Set, Flat Head Phillips Screw Assortments, M1.4/1.7/2/2.5/3 Countersunk ssd Screws Accessories for SSD Toshiba DELL Sony Samsung: Electronics
Pin male header	13	0.0899	1.15	100pcs Male Header Pins, Lystaii Straight Single Row 40 Pin 0.1 Inch (2.54 mm) Male Pin Header Connector PCB Board Pin Connector Electronic Component Raw Materials:Amazon.com: Industrial & Scientific

Both PCBs were ordered with 2 layers, a board thickness of 1.6 mm, an Outer copper weight of 1 oz, and a base material of FR-4.

Another detail that must be noted is the frequency of the LoRa at the location where the data logger network will be used. At the location where this data logger network was developed (USA), it is required to use frequencies between 902 and 928 MHz. The LoRa frequency will change the product specifications of the LILYGO TTGO and RFM LoRa boards.

Lastly, if the remote databases are planned to be used, it is necessary to count the charges of the web server that is hosting the API and the hosting cost of the MongoDB database. For hosting the API, a simpler server can be used since the computational requirements are not high. There are some options starting from US$ 5 per month. For the MongoDB database, a low amount of data can be stored for free in some web services.

## 6 Build instructions

**Fig. 5:**
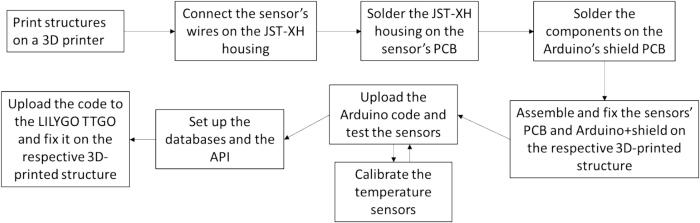
Summarization of required steps to assembly the proposed hardware.

Fig. 5 provides a workflow on how to assembly the proposed hardware, summarizing the main steps needed for the implementation:

### 6.1 Structures

**Fig. 6:**
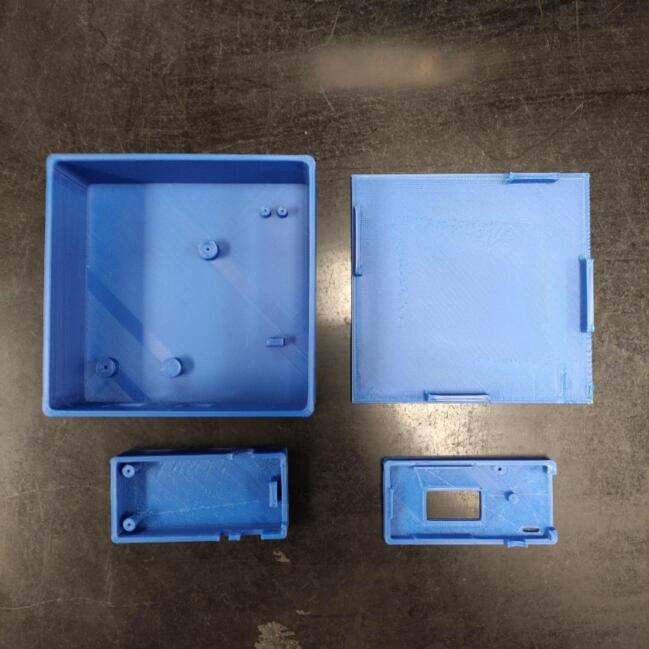
3D printed structures used for building the sensor.

Start the building process by 3D printing the necessary structures present in Table 5. These structures can be printed using Polylactic Acid (PLA) material, 20 % gyroid infill, and 0.2 mm height (Fig. 6, shows all the printed structures).

### 6.2 Data loggers

**Fig. 7:**
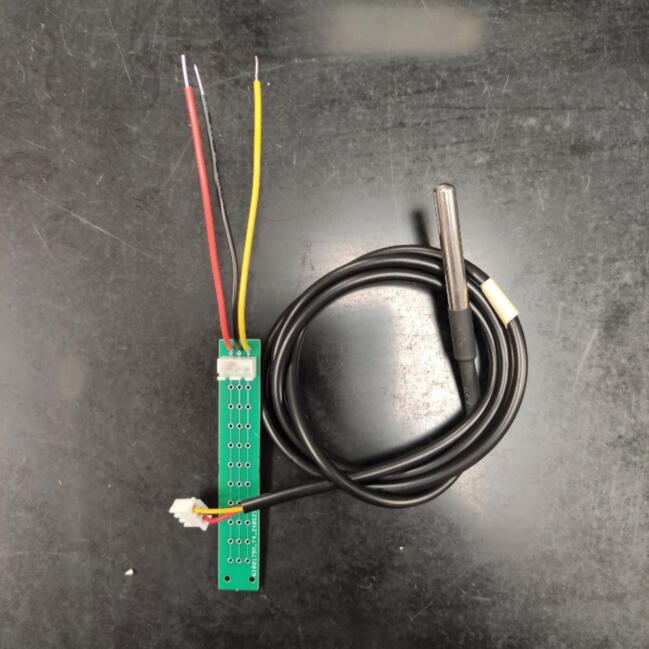
Connecting the Temperature sensors to the sensor’s PCB. All sensors’ terminals must be in the same PCB trail.

The first step to assembling the Data Loggers is connecting the Temperature sensors to the Sensor’s PCB. To perform this step, it is necessary first to connect the Temperature sensor’s wires to the 3-pin female JST-XH housing. During this step, the usage of a hand crimper tool is recommended. Always remember to assemble the crimped wires in the JST-XH housing in the same order to avoid misconnections. We recommend following the same order in the PCB text layer: VCC, GND, and Signal. After this step, it is necessary to solder the 3-pin male JST-XH housing in the Sensor’s PCB to connect the sensor to the PCB. Next, strip the tips of the 3 sets of copper wires and solder them in the Sensor’s PCB in the same order as stated in the PCB text layer. The output of this first step can be visualized in Fig. 7.

**Fig. 8:**
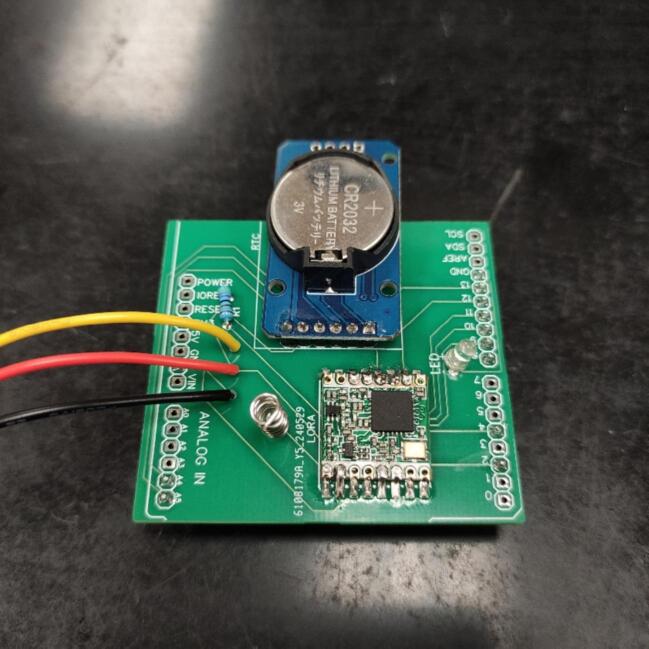
Arduino’s Shield PCB appearance after soldering all components.

The next step is to solder the components to the Arduino’s shield PCB. To save time, solder the Pin Male Header only to the 3 V3, GNDs, A4, A4, 2, 4, 8, 9, 10, 11, 12, and 13 pads of the PCB. Please note that it is necessary to solder them facing the opposite side of the writings because they will be used to connect to the corresponding terminals in the Arduino Uno. Then use the PCB text layer as a guide for soldering the other components. The connection of the sensors’ PCB to the Arduino shield PCB requires more attention. This can be done by simply soldering the wires connecting the two PCBs to the appropriate pads. Another alternative is to use a 3-pin JST-XH package. Note that they must follow the same order: the wire from the VCC in the sensors’ board must connect to the VCC pad indicated in the Arduino shield PCB, and the same for the other two (GND and Signal). To build the antenna, strip the 7.8 cm 1 mm copper wire, and make a coil. Next, solder it to the pad above the LoRa. The end of these steps can be visualized in Fig. 8.

**Fig. 9:**
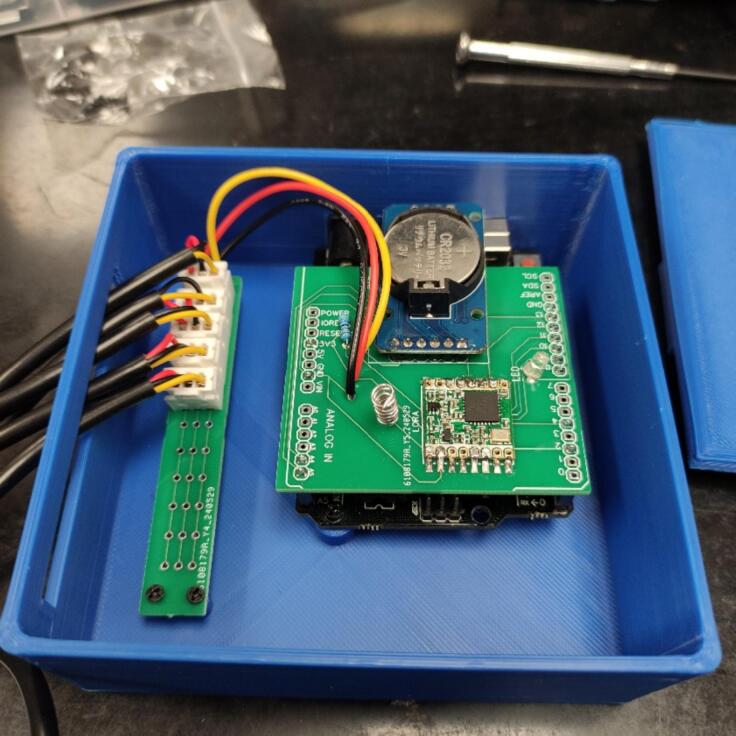
Data logger appearance after is fully assembled.

Next, place the assembled components into the 3D printed structures. Use the M1.7 screws and the M2 screws to secure the Sensor Shield and the Arduino Uno, respectively. Attach the Arduino Shield PCB to the Arduino Uno by connecting the respective pads from the Shield to the Arduino. Remember to feed the temperature sensor’s wire through the housing cavity before connecting it to the sensor PCB. The end of this step can be seen in Fig. 9.

Before uploading the Data Logger’s Arduino (.ino) code using the Arduino IDE (which can be downloaded from the internet free of charge), it is necessary to pay attention to four important details:

• In line 4 it is necessary to set the id of the data logger. This id will later tell us which device the data comes from.
• In line 5, it is necessary to set the number of sensors being used.
• In line 6, it is necessary to set the interval between readings, in seconds.
• In line 7, it is necessary to set the frequency of the LoRa module you are using.
• In lines 8 and 9, it is necessary to set the calibration values for the sensor reading with the model inclination and coefficient, respectively;
• The code to configure the RTC must be uploaded twice: once with line 59 uncommented and once with it commented.

After uploading the code, it is necessary to label the sensors. A tip is to set the interval between seconds to one second for this step. Use ice cubes, a soldering iron, or hot breath to individually cool or heat the temperature sensors, and check the Serial Monitor for the readings. Label the physical sensor with the number whose reading is changing in the Serial Monitor.

If the white LED turns on during uploading or powering up the dataloggers, there is an error in the datalogger. This could be RTC not detected, LoRa board not detected, or the number of sensors found by the logger is different from the number configured in the algorithm. The Serial Monitor will inform you which problem needs attention. To fix these errors, check that these devices are not shorted, are soldered correctly, or are working properly.

### 6.3 Temperature sensor calibration

A necessary step is to calibrate the temperature sensor to ensure consistency and accuracy. To perform it, Koestoer et al. [27] described a method to calibrate the DS18B20 temperature sensor. We have followed the same procedure as previously described by Koestoer et al. [27] but with slight modifications. We have changed the oil using water and a clinical probe thermometer already calibrated to obtain the temperature values. The sensor values were obtained using the serial monitor, and the values from the thermometer were annotated. Later, we have used a simple regression line to correlate the values and obtain a correction factor to be used in the Arduino code.

### 6.4 Setting databases and API

The first step in setting up the local database is to create a.txt file on the microSD card. Connect the microSD card to a computer and create a new text file with the extension “.txt” in the main folder. Inside the file, text and save a line with the following text “iD, Reading, DateTime”. The previous text will be the header for the stored data.

To set up the remote database, it is necessary to configure a remote MongoDB cluster. There are several options for creating the MongoDB cluster, such as Amazon Web Services (AWS), Microsoft Azure, Google Web Services (GWS), and MongoDB Atlas. The researcher must analyze which one best fits the project budget. After selecting the service and setting up the environment, researchers need to create a MongoDB cluster, in this remote cluster, they need to create a database and a collection within it. Note that to access the database from different IP addresses, it is necessary to allow this in the configurations. Store the connection string to access the database under the name of the database and collection. They will be used in the API configuration.

The Python code described in Table 2 is the algorithm that must be deployed to the web service as an API. The web services to which this API can be deployed are the same as those for MongoDB Cluster, with the exception of MongoDB Atlas. To facilitate deployment, we have already created a docker file in the Python code folder and repository, which is necessary to create a container that will run the API. Before uploading, however, some modifications are required. In lines 12, 13, and 14, the text between the quotes must be replaced with the access string, the database name, and the collection name, respectively. After these modifications, the algorithm is ready to be deployed as an API to the Web services. The server used to deploy the API will have a unique domain, this domain will be used in the LILYGO TTGO to send the data to the API and in the digital tool to pull the data from the database.

### 6.5 LILYGO TTGO

In this study, we have provided three different options of codes for the LILYGO TTGO: both remote and local data storage, local data storage only, and remote data storage only. Please, select the one that best fits your project for uploading to the board. Before uploading the LILYGO TTGO receiver code to the board, it is necessary to pay attention to some details:

• − In line 4, it is necessary to change the name “test.txt” in the function “D.open(“/test.txt”, FILE_APPEND)” to the name previously set for the.txt file in a local database on the microSD card.
• In line 5, the used LoRa frequency has to be set.
• Finally, change the variables in the secrets.h file. The variable “ssid” is the name of the WiFi network to which the LILYGO TTGO will connect; the “password” is the password for this network; and “serverUrl” is the URL of the API set previously. Note that the “/store” endpoint must be retained to inform the API algorithm that the data is being sent for storage.

Upload the algorithm to the LILYGO TTGO without the microSD card inserted in the board, otherwise a connection error will occur.

**Fig. 10:**
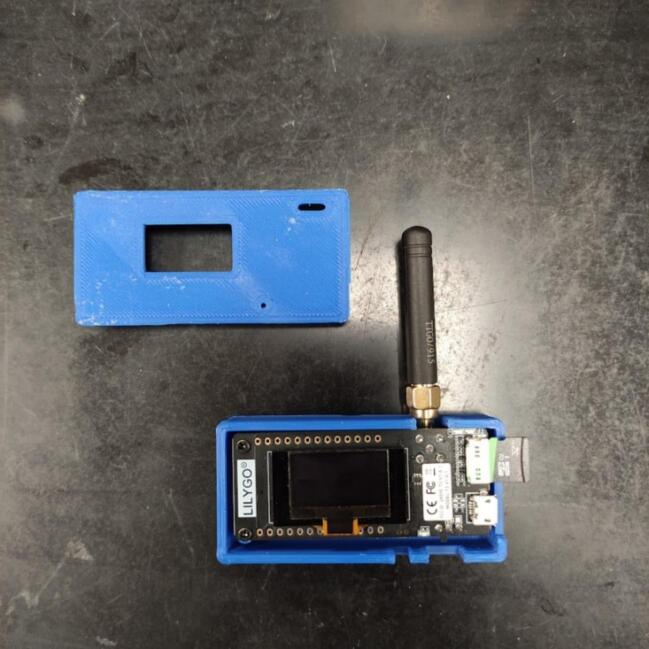
Appearance of the LILYGO TTGO after is assembled and fixed in the 3D-printed structure.

Lastly, use the M1.7 screws to fix the LILYGO TTGO in the 3D-printed structure and fit the cover according to the structure format (Fig. 10).

## 7 Operation instructions

To start using the data loggers’ network, insert the microSD card in the LILYGO TTGO and, using a cable, connect it to a power source, turning it on. Moving to the data loggers’ network, place all the temperature sensors in the desired locations. Next, connect the data loggers to the power source through the appropriate cable. It is highly recommended to wait a few moments to connect different data loggers to the power source since the data is instantly sent to the LILYGO TTGO. Connecting two or more data loggers to the power source at the same time can overload the connection, potentially leading to errors.

### 7.1 Digital tool

Using RStudio [28] or another Integrated Development Environment (IDE) that can run R computer language, open the runApp.R file. Please, do not change, move, or delete the other folder files. Next, run the lines 2 to 5 to install the required packages and its dependencies. This step may take time. After the packages are installed, run line 8 to reference the function where the shiny app is. Lastly, modify and run line 8 by inserting the function arguments:

• “file.source” argument is the data source: “txt” from the microSD card or “NoSQL” from the remote database.
• If your data is coming from the microSD card, set the “file.path” argument to the path of your.txt file.
• If your data is coming from the remote database, set the “GET.API” argument to the server URL + “/obtain.” This server URL is the same one used in the LILYGO TTGO in the variable serverUrl in which you can access your server, just changing the endpoint “/store” to “/obtain”.

Please note that in the tool, the “block” is related to the iD of the datalogger set by the researcher while uploading the code to the Arduino.

## 8 Validation and characterization

### 8.1 Sensor calibration

In this study, we used five DS12B20 to obtain the correction factor for all the other sensors used. We used R [26] and the packages metrica [29] and ggplot2 [30] to obtain both the calibration metrics and curve graph. For metrics, the root mean square error (RMSE), mean average error (MAE), and mean average percentage error (MAPE) were calculated to compare values obtained from the sensors against those from the thermometer. Finally, a linear model was fitted to obtain the model inclination and coefficient to be inserted in the Arduino code.

**Fig. 11:**
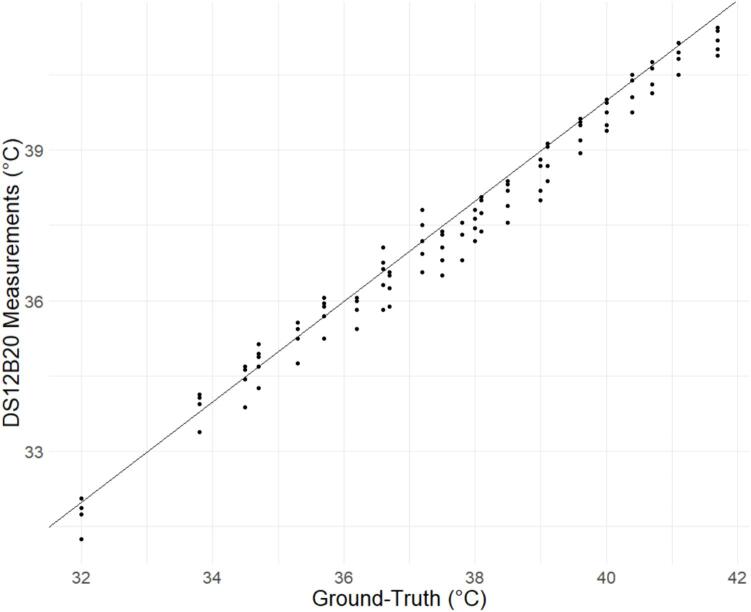
DS18B20 sensors reading vs. Ground-Truth during sensor calibration.

Fig. 11 shows the calibration curve graph for the ground truth obtained with the clinical probe thermometer and the measures recorded by the DS12B20 sensors. The DS12B20 temperature sensor proved to have a high accuracy with an RMSE of 0.41°C, an MAE of 0.32 °C, and a MAPE of 0.86 % (Table 4), proving the reliability of this sensor. The model inclination and coefficient obtained were −0.48 and 1.02, respectively. These are the values to be inserted in lines 8 and 9 of the Arduino code from Section 5.2. Data Loggers.

**Table 4:** Metrics obtained during sensor calibration.

RMSE (Celsius degrees)	MAE (Celsius degrees)	MAPE (%)
0.416	0.324	0.86

### 8.2 Validation experiment

To validate the temperature data logger network, a simple germination test was conducted in a growth chamber under controlled conditions. The chamber was maintained at 28 °C for 10 h and 22 °C for 14 h, with 60 % relative air humidity. Five pots (540.74 cm^3^ volume, 4.75 cm radius, and 8.5 cm height) were filled with commercial garden substrate, and another five pots of the same volume were filled with soil. The pots were randomly placed within the growth chamber, and two corn seeds were sown in each pot at a depth of 1 cm in each pot. The pots were manually irrigated daily with 40 mL of tap water daily at 11 am, following the manual and data logger measurements.

Two data loggers were used: one with five temperature sensors for the substrate-filled pots and another with five sensors for the soil-filled pots. Each sensor was inserted into a single pot at a depth of 4 cm. Both data loggers were programmed to record temperature measurements every thirty minutes, and a commercial probe thermometer was used to manually measure the temperature of each pot every two hours, from 8 a.m. to 6 p.m. Temperature was manually determined at the same time as the data loggers were collecting soil temperature records. The probe thermometer was inserted into the soil at the same depth as the data logger temperature sensors. The LILYGO TTGO was placed outside the growth chamber to test the ability of the radio signal to transmit data over distance and across physical barriers. The data loggers collected data for 3 days, sufficient time for the corn to emerge under these conditions.

**Fig. 12:**
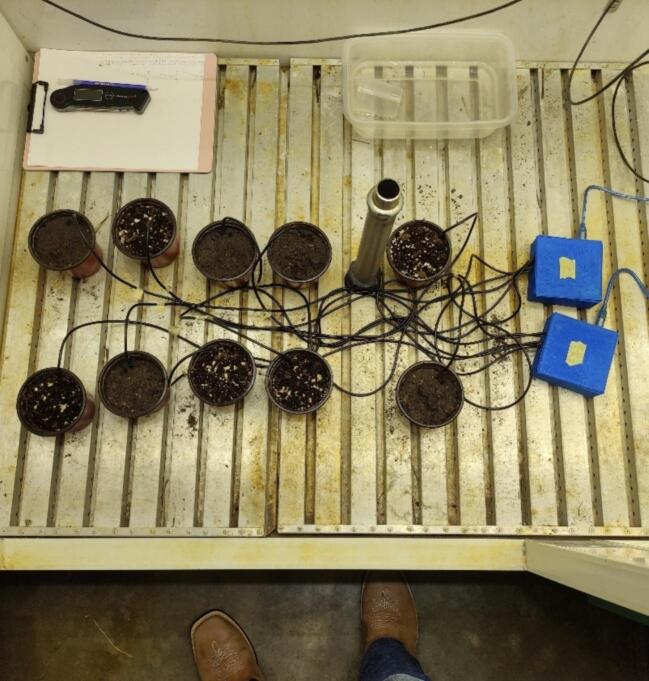
Disposition of pots, data loggers, and temperature sensors during the validation.

Fig. 12 shows the arrangement of the pots, data loggers, and temperature sensors in the growth chamber. This setup allows for comprehensive monitoring and validation of the temperature data logger network under controlled environmental conditions. The comparison of manual probe thermometer readings and data logger measurements helps to verify the accuracy and reliability of the network for future research applications.

**Fig. 13:**
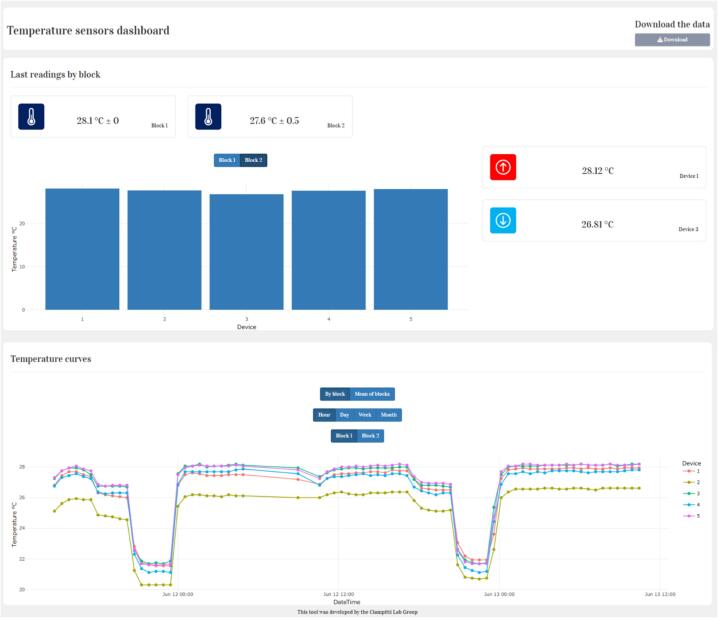
Screenshot of the digital tool while performing the validation experiment.

The digital interactive decision tool was utilized to monitor the growth chamber remotely and to visualize nighttime temperatures. Fig. 13 displays a screen capture obtained during the validation experiment. The interface includes a header titled “Temperature Sensors Dashboard” and a button to download the data as a CSV format for further analysis and evaluation. Below the header is a section labeled “Last Readings by Block,” featuring cards that show the mean of the last reading for each block and the mean–variance. Additionally, there is a bar graph illustrating the last readings of each sensor and two cards displaying the minimum and maximum last readings along with their respective devices. The digital interactive decision tool also features a section for historical temperature curves, which can be displayed by block or by the mean readings of each block. The data can be grouped by hour, day, week, or month for detailed analysis. When the DS18B20 sensor has a reading error, it stores the reading as −127 (an impossible value for this sensor). The proposed digital tool already deals with this error by removing it from the database.

After three days of data collection, the CSV file downloaded from the digital tool was used for data analysis. The CSV file has the columns “Group”, relative to the data logger Id, “DateTime” relative to the date and time (MM/DD/YYYY HH:MM) that the reading was done, “Device” relative to the sensor Id within the data logger, and “Reading”, relative to the temperature value (in Celsius). First, a comparison with the probe thermometer data was performed, calculating the RMSE, MAE, and MAPE using the “metrica” package in R [29]. A two-sample *t*-test was then conducted in R using only the data logger measurements to determine if there was a significant temperature difference between the substrate and soil. Finally, a comparative analysis between block 1 and block 2 was plotted using the ggplot2 package in R [30]. Since the data is in this format, simple grouping and filtering functions were necessary to manipulate the data logger data and compare it to the ground-truth data.

The results showed an RMSE of 1.65 °C, an MAE of 1.55 °C, and a MAPE of 6 % between manual and data logger measurements (Table 5). These results are most likely due to the combination of two factors: minor timing differences in data collection between the data logger recording and the manual collection and potential heat exchange when opening the growth chamber. For the second point, since the growth chamber had to be kept open for manual temperature collection, the heat exchange between the outside and inside may have affected the readings. Heat exchange when opening and keeping the doors open has been widely discussed in the literature [31], [32], [33], strengthening this possibility.

**Table 5:** RMSE, MAE, and MAPE metrics obtained from the comparison of temperature data obtained by the manual sampling using a probe thermometer and data logger data collections.

RMSE (Celsius degrees)	MAE (Celsius degrees)	MAPE (%)
1.65	1.55	6.00

The *t*-test indicated that there was no significant difference in temperature between the soil and the substrate at 5 % probability due to the p-value being equal to 0.4393 (Table 6). The mean temperature obtained between groups 1 (commercial substrate) and 2 (soil) was very similar, with 26.52 and 26.43, respectively. These results showed that the physical properties of the soil between these two do not present any difference in the internal temperature for seedling germination. In Akter et al. [34], different soil textures and different temperatures were tested, and it was observed that the soil temperature varied depending on the texture. Since the substrate and the soil in our study had similar textures, the non-significance of the temperature obtained may be explained by the findings of Akter et al. [34].

**Table 6:** P-value and means obtained from the two-sample*t*-test comparing temperature data collection from pots with substrate (group 1) and soil (group 2).

p-value	Mean group 1 (Celsius degrees)	Mean group 2 (Celsius degrees)
0.4393	26.52	26.43

**Fig. 14:**
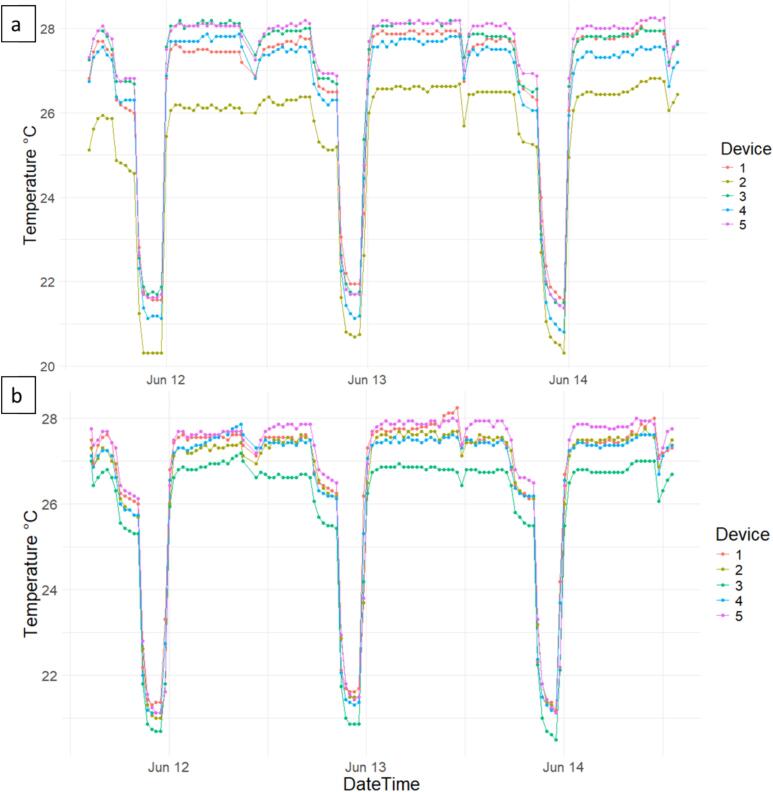
Historical temperature readings obtained during the validation experiment using the proposed data loggers for commercial substrate (a) and soil (b).

Since the temperature inside the growth chamber varied during the night, and this temperature was not recorded due to time constraints and lack of available help, it is vital to verify the behavior of the sensors during this period. Fig. 14 shows the temperature readings for the entire validation period. It can be seen that the data logger readings successfully tracked the temperature changes inside the growth chamber. The distant readings of sensor 2 in Fig. 14a and sensor 3 in Fig. 14b can be either by i) proximity to colder regions within the growth chamber or ii) sensor with slight defect.

### 8.3 Characterization

When analyzing the total cost of the validation, including 10 sensors in two data loggers and one month of web service, the total cost was roughly $102. In contrast, the closest commercial product − a 12-channel data logger with both remote and local storage − costs $280 (not including sensors). For two blocks, the cost would be US$560, not including sensors. Thus, the data logger network presented in this study represents only 18 % of the cost of the closest commercial product. Using only a local database, the validation cost drops to US$97, compared to US$238 for the closest commercial product, or 232 % of the cost of our data loggers. Similar economic advantages when developing low-cost data logging systems were reported by Fuentes et al. [19], developing a low-cost autonomous data logger for photovoltaic systems. The aforementioned authors reported a minimal economy of 300 % over the closest commercial version. When developing a low-cost multi-channel data logger, Saha & Hossain [35] presented hardware costing US$30 (2006 values), which was stated to have a reduced cost compared with commercial versions. The data logger developed by the aforementioned authors only stored data locally and did not have a network, allowing readings from multiple locations.

Regarding energy consumption, since this hardware was designed for indoor usage (greenhouses and growth chambers), therefore locations where power saving is not a problem, we did not assess its practical power consumption. To make users aware of the proposed network power consumption, the theoretical power consumption was calculated using the datasheets of the used hardware. Table 7 shows the results obtained. The LILYGO TTGO has a power consumption varying from 10 to 14 mAh, and the data loggers without the DS18B20 temperature sensors (with Arduino Uno, RFM96 LoRa board) have an initial power consumption of 25.6 and 134 mAh in sleep and active mode, respectively. For each DS18B20 temperature sensor within the data logger, an additional 0.00075 and 1 mAh must be added to the power consumption in sleep and active mode, respectively.

**Table 7:** Theoretical power consumption for the LILYGO TTGO and the proposed data loggers in sleep and active modes.

Hardware	Power consumption (mAh)
LILYGO TTGO LoRa32	10–14
Data logger with N temperature sensors in sleep mode	25.6 + N * 0.00075
Data logger with N temperature sensors in active mode	134 + N * 1

We have encountered six major limitations with this hardware. First, the interference of the LoRa signal, receiving empty measurements from the data loggers. Orfanidis et al. and Polak et al. [36], [37] have also investigated similar interference of the LoRa signal, which was mainly attributed to signals from other sources using the same frequency band. Second, the connection of the LILYGO TTGO to the WiFi network. During the validation step, we have noticed that this board connects better to a 2.4 GHz WiFi network. This limitation is because the ESP8266, the embedded component responsible for the WiFi connection in the LILYGO TTGO board, operates at a 2.4 GHz frequency [38]. Third, when connecting a new sensor, it is necessary to re-label all sensors. The Arduino recognizes the sensors by their hexadecimal addresses and sorts them out by generating a sequence. If the newly connected sensor has a hexadecimal address smaller than or in the middle of the other addresses, it will take its position in the array, changing the labeled order. Fourth, the projected structure is not weatherproof, which limits it to indoor use. This limitation reduces the usability of the hardware in different studies [26]. Fifth, the sensor reliability and durability, long-term duration, should be further evaluated under different conditions. Future studies can focus on moving this sensor to field settings with the goal of testing of environmental factors such as different soil types, equipment layout, and moisture conditions.

A large limitation is adjusting the time gap between readings, which can conflict with the number of sensors within a data logger and the number of data loggers in the network. Suppose the time between readings is too short for the number of sensors in one data logger. In that case, the Arduino will attempt to perform the next reading while still performing the previous, generating reading problems. The same will occur with numerous data loggers in the network with a short interval between measurements. The last data logger will send information to the LILYGO TTGO simultaneously with the first one, generating problems while storing data. The solution found while performing tests was to empirically set the time intervals considering the desired number of sensors within a data logger and the number of data loggers in a network.

Future studies for this hardware should aim at modifications that would allow it to be used under field settings. This would require i) develop a weatherproof structure by using a different material for the 3D printed structure, ii) additional insulation for the sensor inputs to keep water and other contaminants out of the system, iii) accurately measuring the power consumption to design a battery capacity for extended periods of time, and iv) designing alternative methods of powering the device such as solar panels and small wind turbines. An alternative to address the first aim is to use the same method as stated by Afshar & Wood [39] when designing weather resistant 3D printed structures. For the third and fourth objectives, a similar approach was used by Bayhan & Turhan [40], to design a solar-based data logger, which could be used as a model. However, for the fourth goal, a combination of solar and wind power generation could be used, as exemplified by Hossain et al. [41], or the use of small wind turbines only could be explored, as explored by Wang et al. [42].

In summary, a low-cost network of multi-channel data loggers has been developed for indoor temperature measurements. This network is capable of storing data locally, on a microSD card, and remotely in a NoSQL database. The network developed has an initial cost of US$ 72 (2024 values), and its cost comparison with commercial versions decreases as the experimental size increases. A single data logger can receive data from up to 24 temperature sensors, while most commercial versions have a maximum of 12 sensors. In addition, due to the LoRa connection between the data loggers and the central unit (LILYGO TTGO), the loggers can be installed apart from each other without connecting cables. The validation experiment performed in a growth chamber demonstrated the reliability of the network to read and store temperature data locally and remotely, even when placed away from the central station. Lastly, the digital tool allowed the visualization of past readings, successfully splitting the data by the data loggers and temperature sensors, exporting the data in CSV format for further analysis. This hardware has the potential to help researchers, especially (but not limited to) soil temperature experiments in greenhouses or growth chambers.

### CRediT authorship contribution statement

**Gustavo N. Santiago:** Conceptualization, Data curation, Formal analysis, Investigation, Methodology, Software, Validation, Visualization, Writing – original draft. **Ignacio Ciampitti:** Writing – review & editing, Validation, Supervision, Resources, Project administration, Funding acquisition.

## Declaration of competing interest

The authors declare that they have no known competing financial interests or personal relationships that could have appeared to influence the work reported in this paper.
